# Preoperative prediction of pituitary neuroendocrine tumor invasion using multiparametric MRI radiomics

**DOI:** 10.3389/fonc.2024.1475950

**Published:** 2025-01-09

**Authors:** Qiuyuan Yang, Tengfei Ke, Jialei Wu, Yubo Wang, Jiageng Li, Yimin He, Jianxian Yang, Nan Xu, Bin Yang

**Affiliations:** ^1^ Department of Medical Imaging, The Second People’s Hospital of Dali Prefecture, Dali, China; ^2^ Department of Medical Imaging, Yunnan Cancer Hospital, Kunming, China; ^3^ Medical Imaging Center, The First Hospital of Kunming, Kunming, China; ^4^ Department of Radiology, Air Force Medical Center, Air Force Medical University, Beijing, China

**Keywords:** pituitary neuroendocrine tumor, radiomics, magnetic resonance imaging, cavernous sinus, prognosis

## Abstract

**Objective:**

The invasiveness of pituitary neuroendocrine tumor is an important basis for formulating individualized treatment plans and improving the prognosis of patients. Radiomics can predict invasiveness preoperatively. To investigate the value of multiparameter magnetic resonance imaging (mpMRI) radiomics in predicting pituitary neuroendocrine tumor invasion into the cavernous sinus (CS) before surgery.

**Patients and methods:**

The clinical data of 133 patients with pituitary neuroendocrine tumor (62 invasive and 71 non-invasive) confirmed by surgery and pathology who underwent preoperative mpMRI examination were retrospectively analyzed. Data were divided into training set and testing set according to different field strength equipment. Radiomics features were extracted from the manually delineated regions of interest in T1WI, T2WI and CE-T1, and the best radiomics features were screened by LASSO algorithm. Single radiomics model (T1WI, T2WI, CE-T1) and combined radiomics model (T1WI+T2WI+CE-T1) were constructed respectively. In addition, clinical features were screened to establish clinical model. Finally, the prediction model was evaluated by ROC curve, calibration curve and decision curve analysis (DCA).

**Results:**

A total of 10 radiomics features were selected from 306 primitive features. The combined radiomics model had the highest prediction efficiency. The area under curve (AUC) of the training set was 0.885 (95% CI, 0.819-0.952), and the accuracy, sensitivity, and specificity were 0.951,0.826, and 0.725. The AUC of the testing set was 0.864 (95% CI, 0.744-0.985), and the accuracy, sensitivity, and specificity were 0.829,0.952, and 0.700. DCA showed that the combined radiomics model had higher clinical net benefit.

**Conclusion:**

The combined radiomics model based on mpMRI can effectively and accurately predict the invasiveness of pituitary neuroendocrine tumor to CS preoperatively, and provide decision-making basis for clinical individualized treatment.

## Introduction

Pituitary neuroendocrine tumors (PitNETs) are common intracranial neuroendocrine tumor, with a prevalence as high as 20% on autopsy and imaging studies, have shown a significant upward trend in recent years (1–3). Previous studies have generally considered PitNETs to be a common intracranial benign tumor (4), however, a portion of PitNETs are invasive, their benign nature is not so apparent, especially when the diameter is greater than 10mm, and their expansive growth can infiltrate into the surrounding structures. This emphasizes the importance of a multidisciplinary and comprehensive treatment plan (5), and how to predict its invasiveness preoperatively is exactly the problem we need to solve. Research has reported that nearly 25–55% of PitNETs (diameter >10mm) are invasive, and often invade adjacent structures, especially cavernous sinus (CS) (4, 6). The internal carotid artery (ICA) is adjacent to the CS, and the invasion rate of PitNETs (diameter >10mm) into the CS is 16% (7), which indirectly affects the success of PitNETs treatment. Clinically, surgery is the preferred treatment for most PitNETs (8). It should be noted, however, that tumor invasion into the CS significantly increases the incidence of surgery-related complications and postoperative mortality (9). Therefore, assisting clinicians in accurately evaluating the degree of PitNETs invasion into the CS before surgery is critical for formulating individualized treatment plans. Relevant studies (10, 11) have reported that if the tumor significantly invades the CS, preoperative adjuvant radiotherapy combined with incomplete tumor resection is required to avoid ICA injury when the tumor is completely resected. Otherwise, tumors without CS invasion require complete resection of the lesion to reduce tumor residue and recurrence. At present, the Knosp grading standard, established by Knosp (12), is the most commonly used method to evaluate the invasiveness of PitNETs, in which preoperative MRI can be used to evaluate the extent of parasellar tumor invasion. It is worth noting that the gold standard for the diagnosis of PitNETs invasiveness the visual observation of the erosion and destruction of the adjacent sella bone during surgery (9). However, these two methods have significant limitations. First, the Knosp grading standard relies on radiologists’ MRI interpretations, which are subjective and closely tied to the work experience of the radiologist, as well as image quality and repeatability. Surgery, as a key treatment for PitNETs, is an invasive procedure that makes it difficult to achieve the unique requirements of preoperative evaluation of tumor invasiveness. Therefore, it is crucial to find a simple, efficient, and accurate method to predict the invasiveness of PitNETs preoperatively, assist in the clinical development of individualized treatment strategies, and achieve precise treatment and long-term management of patients.

The recent development of artificial intelligence (AI) has led to its widespread use across various fields. Radiomics, which can high-throughput mine quantitative image features from medical images, such as computed tomography (CT), MRI, positron emission tomography-CT (PET-CT), and ultrasound (US), use machine learning algorithms to analyze the correlation between features, and establish prediction models to achieve preoperative diagnosis, prognosis prediction, and efficacy evaluation of diseases (13–15), has been extensively applied as an important AI technology in the medical field. Radiomics has been studied and applied to various systemic diseases (16–22), and prediction models have shown high prediction efficiency. PitNETs are common tumor of the nervous system, and Yang et al. (14) summarized the application and research fields of AI technology in detail as it relates to PitNETs. Compared to CT radiomics, MRI radiomics offers better soft tissue resolution, allowing for the display of smaller structures, and provides clearer visualization of structures such as the CS, which can lead to a better diagnosis of tumor invasion into the CS. Its multi-planar imaging and dynamic contrast-enhanced scans are also of significant value in assessing the invasiveness of PitNETs, and its radiation-free nature can meet the clinical needs of PitNETs’ patients for multiple follow-ups. However, there are few reports, on the preoperative prediction of tumor invasion into the CS (6, 23, 24). Liu et al. (23) showed that texture analysis, based on dynamic contrast-enhanced MRI (DCE-MRI), can predict the vascular heterogeneity and invasiveness of PitNETs before surgery. Wang et al. (24) focused on the use of deep learning algorithms to develop automatic saddle area segmentation techniques, and used various tools to extract image features related to invasiveness. These studies, however, only focused on a single MRI sequence, used the prediction of invasiveness as a downstream task, and did not establish an invasive prediction model, lacking clinical guidance. Radiomics based on mpMRI can obtain more potential parameters, which can help evaluate the texture and extent of tumor invasion from different perspectives, can more comprehensively reflect the texture and invasion range of tumors, while single parameter MRI may not provide such comprehensive information. Combining radiomics features of mpMRI sequences can improve the diagnostic performance of PitNETs, have higher efficiency in predicting tumor invasiveness.

The purpose of the present study was to extract radiomics features based on mpMRI to establish a radiomics model to accurately, efficiently, and noninvasively predict the invasiveness of PitNETs to CS preoperatively, and provide guidance for precise individualized treatment.

## Materials and methods

### Patients

The present study was approved by our institutional ethics review committee, and the need for informed consent was waived, due to the retrospective nature of the study.

**Figure 1:**
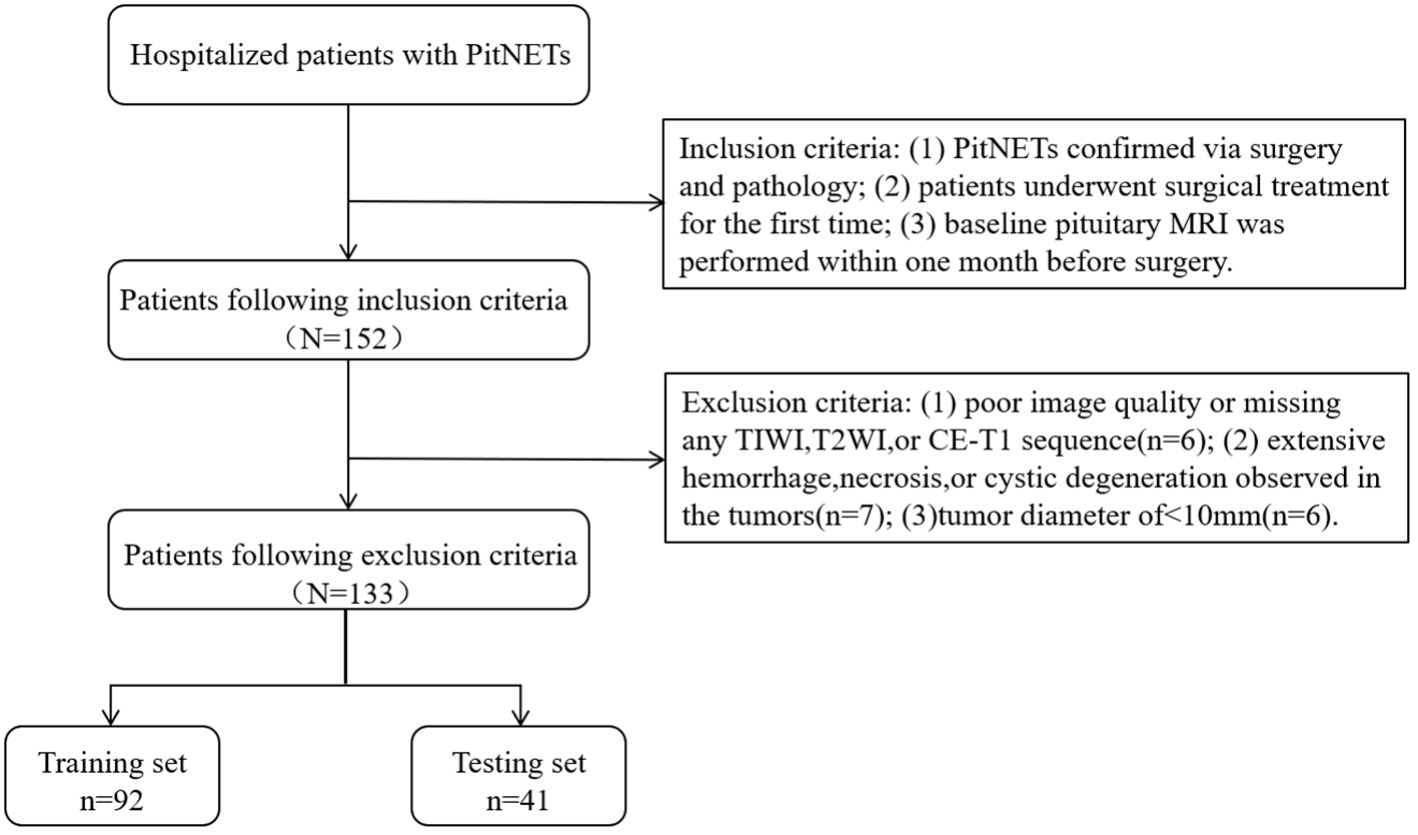
Flow diagram of patient selection and grouping.

This study retrospectively gathered the data of patients with PitNETs who underwent surgical treatment at our institution between January 2012 and December 2020. The inclusion criteria were as follows: (1) PitNETs confirmed via surgery and pathology; (2) patients underwent surgical treatment for the first time; and (3) baseline pituitary MRI was performed within one month before surgery. The exclusion criteria were as follows: (1) poor image quality or missing any T1-weighted imaging (T1WI), T2-weighted imaging (T2WI), or contrast enhanced-T1 (CE-T1) sequence (n = 6); (2) extensive hemorrhage, necrosis, or cystic degeneration observed in the tumors (n = 7); and (3) tumor diameter of < 10 mm (n = 6). For invasive PitNETs, surgeons can observe the tumor’s invasion of the cavernous sinus during the surgical procedure, with all details meticulously documented in the surgical records. After applying the exclusion criteria, a total of 133 patients were included in the present study, after screening according to the nanofiltration criteria (**Figure 1**).

Radiologist 1 (10 years’ work experience) reviewed the surgical records, and had not seen the MRI in advance. Radiologist 2 (7 years’ work experience) reviewed the MRI, and assessed clinical radiographic risk factors (Knosp classification, hemorrhage, cystic degeneration, necrosis, etc.). Patients who met one of the following three criteria were included in the invasive group (6, 11, 23, 26): (1) Hardy classification and staging system grade III–IV or suprasellar extensions C–E, or Knosp grading standard grade III–IV; (2) intraoperative exploration revealed tumor invasion into the adjacent dura mater, sellar floor, and sphenoid or bilateral cavernous sinus; and (3) histological examination of the sellar floor or adjacent dura confirmed tumor cell infiltration. Patients who did not meet the above three criteria were included in the non-invasive group.

### MRI protocol and image acquisition

Imaging sequences included T1WI, T2WI, and CE-T1 sequences, and sellar scans were performed on 1.5- (Signa HDe; GE Healthcare, Waukesha, WI, USA; and Magentom Avanto; Siemens, Erlangen, Germany) and 3.0-T (Signa Pioneer; GE Healthcare, Florence, SC, USA; Ingenia; Philips Medical Systems, Best, The Netherlands; and Vantage Titan; Toshiba, Tochigi, Japan) MRI scanners using a head-phased array coil. The sequence and scan parameters are as follows: Signa HDe equipment parameters, T1 (repetition time [TR], 460 ms; echo time [TE], 12.6 ms), T2 (TR, 3,540 ms; TE, 130.2 ms), layer thickness, 3 mm, layer spacing, 3 mm; Magnetom Avanto, T1 (TR, 400 ms; TE, 8.7 ms), T2 (TR, 3,400 ms; TE, 84 ms), layer thickness, 3 mm, layer, spacing 3.3 mm; Vantage Titan, T1 (TR, 450 ms; TE, 12.0 ms), T2 (TR, 3,000 ms; TE 90 ms), layer thickness, 3 mm, layer spacing, 3.5 mm; Ingenia, T1 (TR, 507 ms; TE, 7.5 ms), T2 (TR, 3,000 ms; TE, 80 ms), layer thickness, 2 mm, layer spacing 2, mm; and Signa Pioneer, T1 (TR, 1,929 ms; TE, 23 ms), T2 (TR, 4,151 ms; TE, 134 ms), layer thickness, 2 mm, layer spacing, 2.5 mm. Contrast-enhanced scans were performed using a high-pressure syringe to inject the contrast agent, gadodiamide, through the elbow vein, with a dose of 0.1 mmol/kg at a rate of 2.5 mL/s, followed by 20 mL of saline to flush the lumen.

### Tumor region of interest delineation

**Figure 2:**
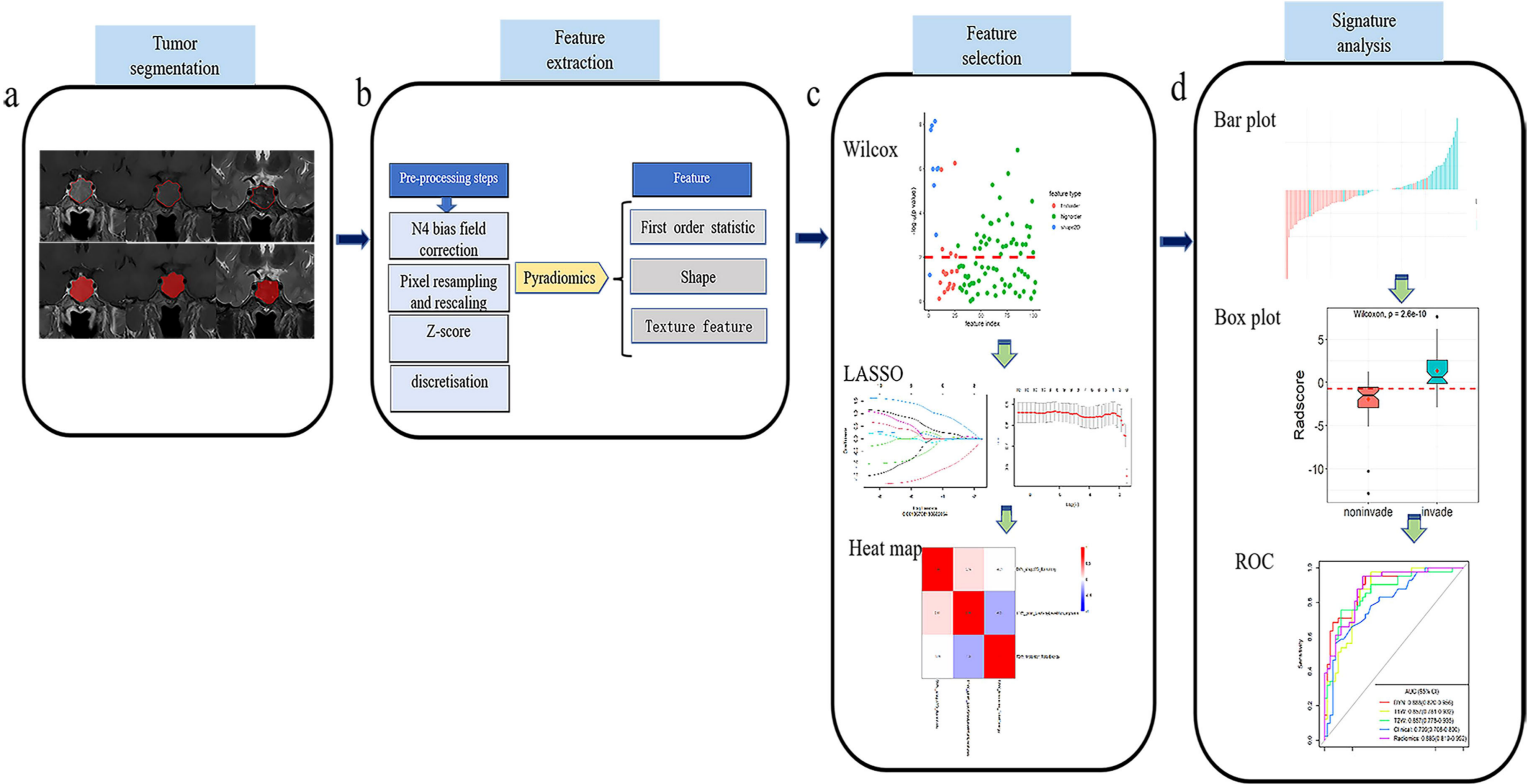
Flow chart of the present study. **(A)** Collect the original T1WI, T2WI and CE-T1 sequence images and use ITK-SNAP software to draw ROI. **(B)** Pyradiomics software was used to extract radiomics features. **(C)** Wilxon rank sum test and minimum absolute contraction sum selection operator (LASSO) are used for feature selection. **(D)** Establish a prediction model, and analyze the prediction efficiency of the best model through the ROC curve.

The T1WI, T2WI, and CE-T1 MRI sequences were exported from our Patient Archive and Communication System (PACS), and saved in Digital Imaging and Communications in Medicine (DICOM) format after anonymization. The images were imported into ITK-SNAP software (version 3.8.0, http://www.itksnap.org). Each ROI was manually delineated at the maximum level of the tumor by radiologist 2, and then reviewed by radiologist 1. When disagreements arise over the delineation of the tumor area, the radiologists conversed with each other to reach a consensus. When delineating the tumor margins, the tumor areas on the T1WI and T2WI images were delineated using the tumor area of the CE-T1 image as the reference baseline (**Figure 2A**).

### Image preprocessing

Because the original MRI scans were acquired on equipment of different manufacturers, models, and field strengths, the diversity of scanning parameters resulted in image heterogeneity. It was therefore necessary to preprocess the original images before extracting the radiomics features, to improve the normalization and standardization of the images. The specific steps were as follows: (1) N4 bias field correction was performed on the image to eliminate low-frequency intensity inhomogeneity; (2) the image voxel space was resampled and adjusted to 1 × 1 × 1 mm^3^ to improve the comparability of the texture features; (3) the z-score method was used to standardize the image gray level, and the maximum and minimum gray levels were limited to three gray standard deviations; and (4) image grayscale underwent discrete transform with a bandwidth set to five (**Figure 2B**).

### Radiomics feature extraction

Quantitative radiomics features were automatically extracted from the ROI of T1WI, T2WI, and CE-T1 sequences using the Pyradiomics platform (27) (version 3.0.1, http://pypi.org/project/pyradiomics). The extracted features included first-order statistical, shape, and texture features (**Figure 2B**). The texture features included a gray-level co-occurrence matrix (glcm), gray-level run-length matrix (glrlm), gray-level size zone matrix (glszm), gray-level difference matrix (gldm), and neighborhood gray-tone difference matrix (ngtdm) (27).

### Radiomics feature screening

The high-dimensional information of radiomics quantitative features is closely related to high-level redundant and irrelevant information, which may lead to overfitting, thereby reducing the performance of machine learning algorithms and seriously affecting the performance of prediction models (28). It was necessary, therefore, to screen the extracted quantitative features before constructing the prediction model. Based on the features extracted from the training set, the feature screening process included the following three steps. First, the Wilcoxon rank sum test was used to retain the characteristics of *P* < 0.01. Second, the Least Absolute Shrinkage and Selection Operator (LASSO) algorithm, based on five-fold cross-validation, was used to remove redundant features from the images. Finally, a multivariate stepwise regression analysis was used, and the feature set with the smallest Akaike information criterion (AIC) was retained (**Figure 2C**).

### Construction and verification of radiomics signature model

From the optimal radiomics features selected, logistic regression (LR) classifiers were used to construct single-sequence (T1WI, T2WI, CE-T1) and combined multi-sequence (T1WI + T2WI + CE-T1) radiomics signature models in the training set. The area under the curve (AUC), accuracy, sensitivity, specificity, and positive and negative predictive values were used to evaluate the performance of the training set model, and then verified in the testing set. Additionally, a receiver operating characteristic (ROC) curve (29) was constructed to evaluate the predictive efficacy of the model. We also established a calibration curve to evaluate the goodness of fit of the prediction model, which was verified in the testing set. The DeLong test was used to compare the prediction efficiency between the models, and the decision curve analysis (DCA) was used to evaluate the clinical net benefit rate (**Figure 2D**).

### Construction and validation of clinical model

Clinical radiological risk factors included sex, age, and maximum tumor diameter. The most relevant clinical features of tumor invasiveness were identified using univariate analysis. Independent predictors were analyzed using multivariate LR regression, and a clinical model was established. The performance of the model was evaluated in the training set, and then verified in the testing set (**Figure 2D**).

### Statistical methods

All statistical analyses were performed using R software (version 4.1.0, https://www.rproject.org). Pearson’s chi-squared or an independent sample *t* test was used to analyze the demographic characteristics. LASSO used the “glmnet” package for analysis, and the Wilcoxon test used the “base” package analysis. ROC curves were plotted using the “pROC” package. Statistical significance was two-tailed, and set at *P* < 0.05. The AUC, accuracy, sensitivity, specificity, and positive and negative predictive values were used to compare and evaluate the predictive efficacy of each model.

## Results

### Clinical characteristics

**Table 1**, a total of 133 patients were included for analysis in the present study, 62 invasive (median age, 49 years; 31 males and 31 females) and 71 non-invasive cases (median age, 43 years; 23 males and 48 females). There were no significant differences in sex, age, or surgical method between the two groups (*P* = 0.059–0.573). There was, however, a significant difference in the maximum tumor diameter (*P* < 0.01), which may be because invasive tumors expand to the surrounding area and have a larger volume.

**Table 1:** Comparison of clinical baseline characteristics of patients with invasive and non-invasive tumors.

	Non-invasive	Invasive	*P*-
Gender (No.)			0.059
Male	23 (32.4%)	31 (50.0%)	
Female	48 (67.6%)	31 (50.0%)	
Age (years)	43.0 (33.5–55.0)	49.0 (37.0–54.5)	0.107
Maximum tumor diameter (mm)	22.0 (15.5–27.0)	33.0 (26.0–37.1)	< 0.001
Surgery method			0.573
Nasopalpebral	63 (88.7%)	52 (83.9%)	
Trans-temporal bone	8 (11.3%)	10 (16.1%)	

Categorical variables are presented as numbers (percentages). Otherwise, median and quartile values are shown. *P* < 0.05 was statistically significant.

In the present study, the patients scanned on 3.0T equipment were used as the training set (n = 92; 51 non-invasive and 41 invasive) to establish the radiomics prediction model, and the patients scanned on 1.5T equipment were used as the testing set (n = 41; 20 non-invasive and 21 invasive) to verify the prediction efficiency of the radiomics model.

### Feature extraction, screening, and analysis

**Figure 3:**
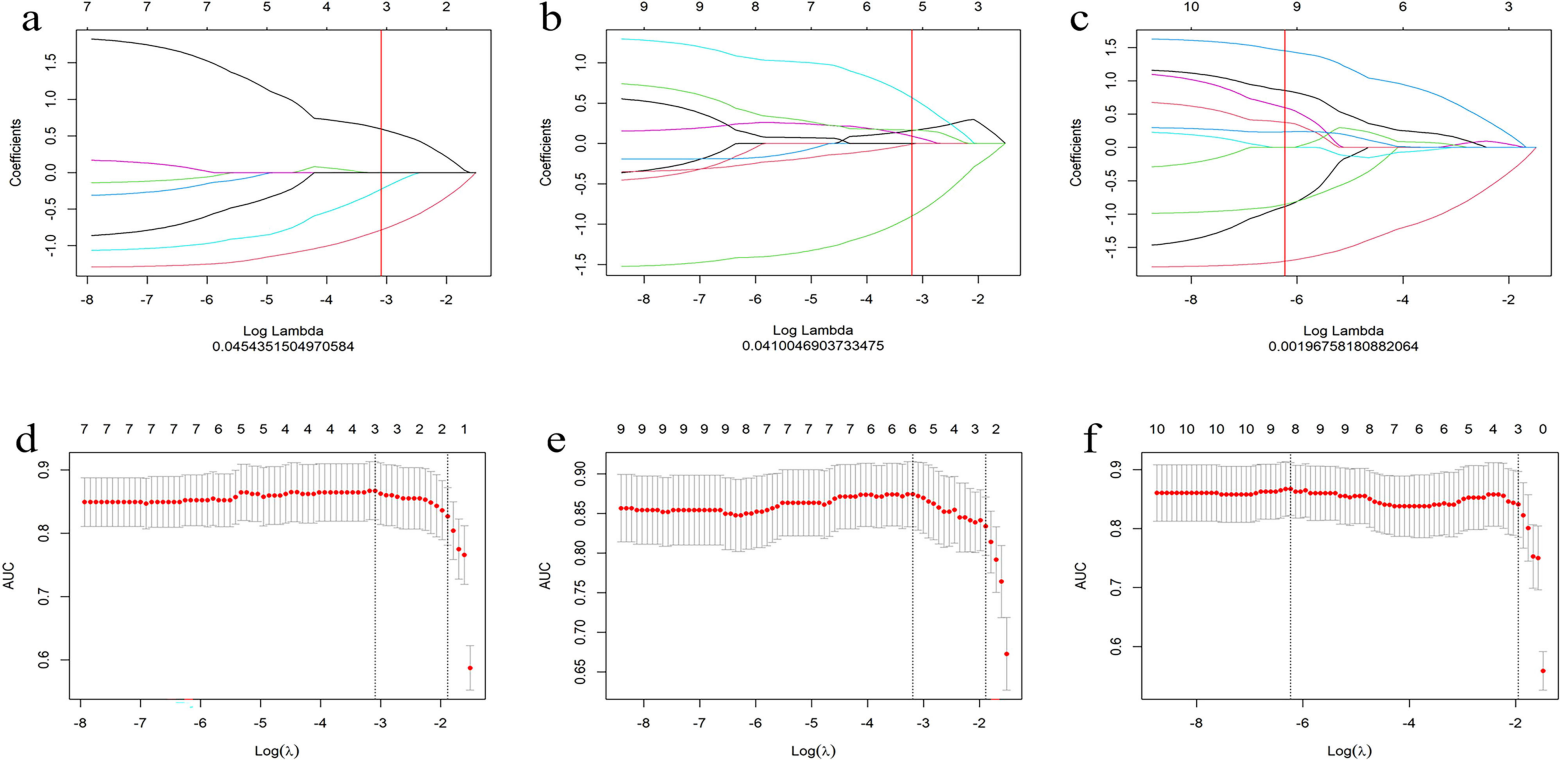
Convergence diagram of characteristic coefficients **(A–C)** of T1WI, T2WI and CE-T1 sequences using LASSO to screen the image group characteristics. Using five-fold cross validation, we screened the most effective histological characteristic map **(D–F)**, and all three sequences obtained the characteristics required by the most simplified model.

**Figure 4:**
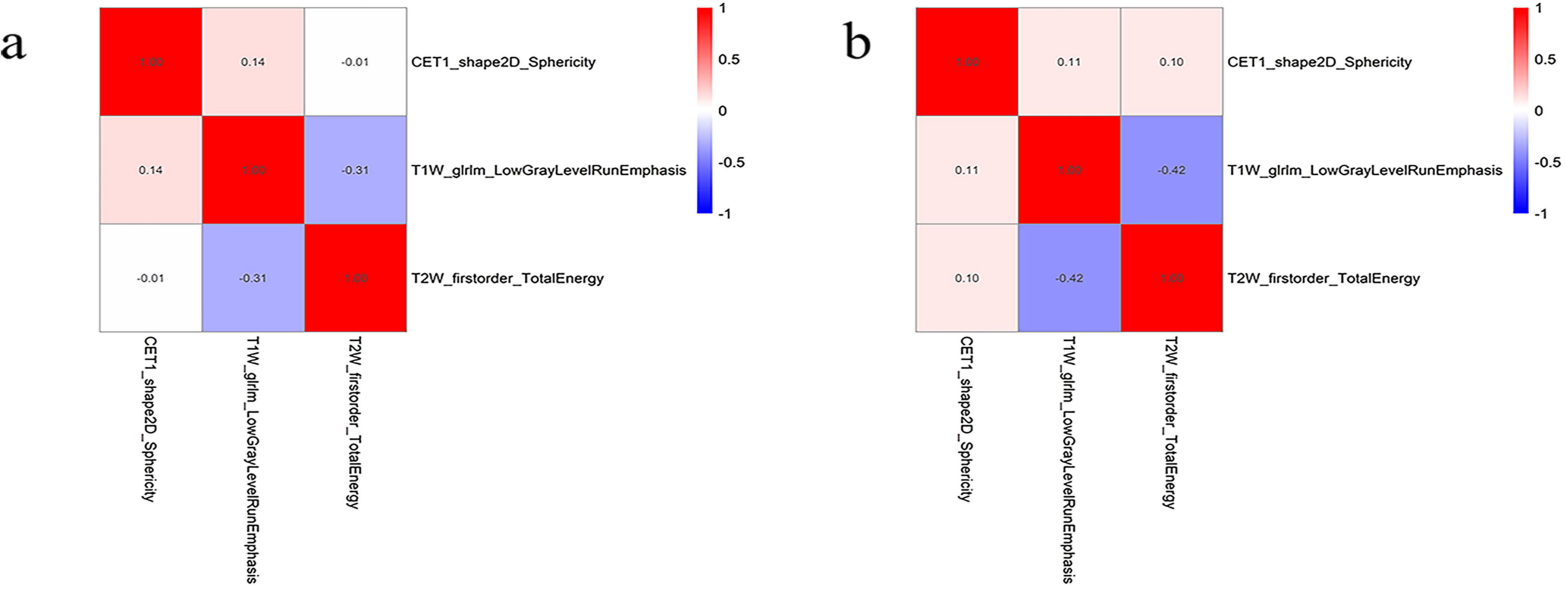
The best feature heat map of the training **(A)** and testing **(B)** sets after feature screening.

A total of 306 radiomic features were extracted from the T1WI, T2WI, and CE-T1 sequences. The radiomics features extracted from single-sequence images included 18 first-order statistic, 9 shape, and 73 texture features (24 glcm, 16 glrlm, 16 glszm, 14 gldm, and 5 ngtdm). Through feature consistency analysis, five-fold cross-validation LASSO regression (**Figure 3**), and multivariate stepwise logistic regression, the best of three features from the T1WI and T2WI sequences respectively, and the best four features from the CE-T1 sequence were selected. After removing the collinear features from the 10 best features, three features were selected to establish a combined radiomics model (**Table 2**). The heat map showed the best characteristics for each sequence (**Figure 4**).

**Table 2:** LASSO analysis screened the most relevant radiomics features to invasiveness.

Best image features	
T1WI	T1W_shape2D_MinorAxisLength; T1W_shape2D_Sphericity; T1W_glrlm_LowGrayLevelRunEmphasis
T2WI	T2W_shape2D_Sphericity; 2W_firstorder_TotalEnergy; T2W_glcm_SumEntropy
CE-T1	CE-T1_shape2D_PixelSurface; CE-T1_shape2D_Sphericity; CE-T1_firstorder_TotalEnergy; CE-T1_glszm_ZoneEntropy
Combined radiomics	CE-T1_shape2D_Sphericity; T1W_glrlm_LowGrayLevelRunEmphasis; T2W_firstorder_TotalEnergy

### Predictive efficacy of clinical model

**Figure 5:**
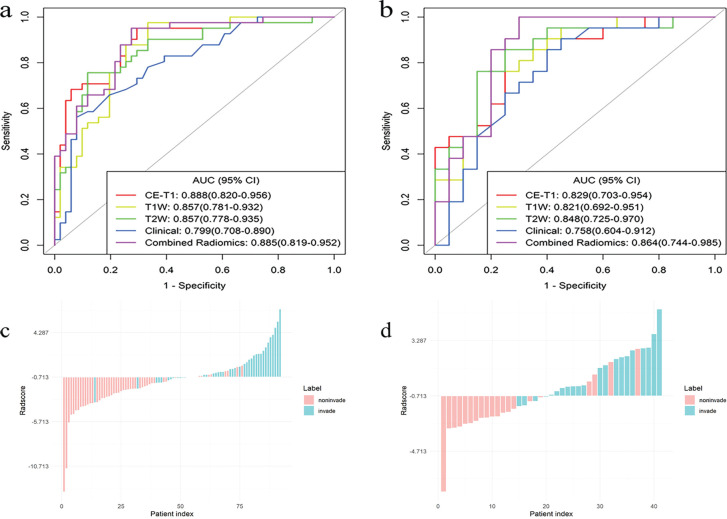
The ROC curves for the single radiomics signature model, clinical model, and combined (CE-T1 + T1WI + T2WI) radiomics signature model, constructed based on an LR classifier in the training **(A)** and testing **(B)** sets, bar diagrams of the training **(C)** and testing **(D)** sets.

Maximum tumor diameter was used as the main clinical risk factor to establish a multivariate regression clinical model. The AUC in the training set was 0.799 (95% confidence interval [CI], 0.708–0.890), and the AUC in the testing set was 0.758 (95% CI, 0.604–0.912) (**Table 3**, **Figures 5A, B**).

**Table 3:** Comparison of prediction efficiency of clinical model, single radiomics signature model, and combined radiomics model.

Model	Performance	AUC (95% CI)	ACC	SEN	SPE	PPV	NPV	Cut-off
Clinical	Training set	0.799 (0.708–0.890)	0.761	0.561	0.922	0.852	0.723	0.583
	Testing set	0.758 (0.604–0.912)	0.707	0.667	0.750	0.737	0.682	
T1WI	Training set	0.857 (0.781–0.932)	0.804	0.976	0.667	0.702	0.971	0.284
	Testing set	0.821 (0.692–0.951)	0.756	0.905	0.600	0.704	0.857	
T2WI	Training set	0.857 (0.778–0.935)	0.826	0.756	0.882	0.838	0.818	0.445
	Testing set	0.848 (0.725–0.970)	0.780	0.905	0.650	0.731	0.867	
CE-T1	Training set	0.888 (0.820–0.956)	0.815	0.951	0.706	0.722	0.947	0.316
	Testing set	0.829 (0.703–0.954)	0.756	0.905	0.600	0.704	0.857	
Combined radiomics	Training set	0.885 (0.819–0.952)	0.826	0.951	0.725	0.736	0.949	0.328
	Testing set	0.864 (0.744–0.985)	0.829	0.952	0.700	0.769	0.933	

AUC, area under curve; ACC, accuracy; SEN, sensitivity; SPE, specificity; PPV, positive predictive value; NPV, negative prediction value; 95% CI, 95% confidence interval.

### Predictive performance of single and combined radiomics signature models

The AUCs of the T1WI model in the training and testing sets were 0.857 (95% CI, 0.781–0.932) and 0.821 (95% CI, 0.692–0.951), respectively. The AUCs of the T2WI model in the training and testing sets were 0.857 (95% CI, 0.778–0.935) and 0.848 (95% CI, 0.725–0.970), respectively. The AUCs of CE-T1 model in training and testing sets were 0.888 (95% CI, 0.820–0.956) and 0.829 (95% CI, 0.703–0.954), respectively. The AUCs of the combined radiomics signature model in the training and testing sets were 0.885 (95% CI, 0.819–0.952) and 0.864 (95% CI, 0.744–0.985), respectively. The accuracy, sensitivity, specificity, and positive and negative predictive values of the four predictive models are shown in **Table 3**. The ROC curve of the prediction model is shown in **Figures 5A, B**. The bar chart in **Figures 5C, D** shows the prediction accuracy of the prediction model in the training and testing sets. The AUC of the CE-T1 model was the highest of the training sets, while that of the combined radiomics signature model was the highest of the testing sets. The performance of the prediction model was comprehensively analyzed and evaluated, and the combined radiomics signature model was selected to achieve the best predictive performance for PitNETs invasiveness. Additionally, the DeLong test showed that the performance of the radiomics model was better than that of the clinical model (*P* = 0.03).

### Model calibration curve and DCA

**Figure 6:**
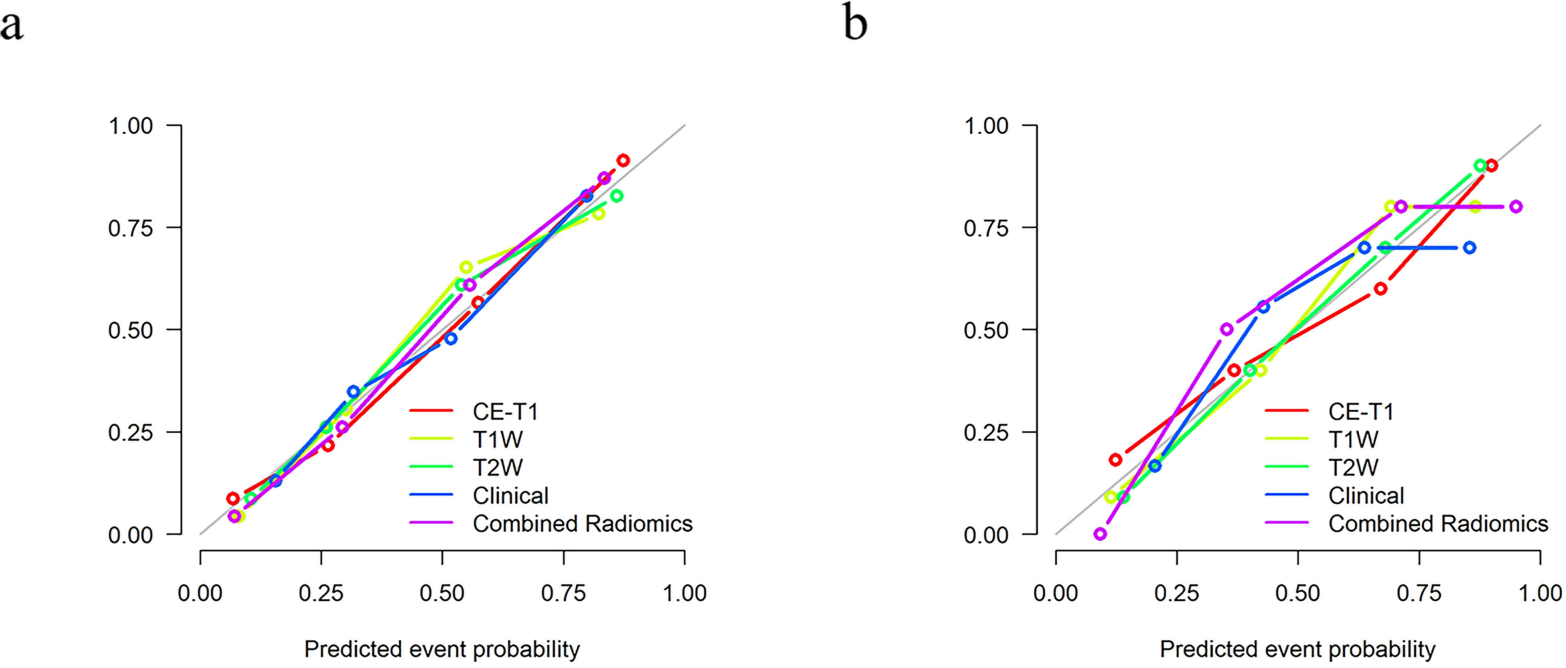
The calibration curve shows that there is a good fit between the prediction model and the actual results in the training **(A)** and testing **(B)** sets.

**Figure 7:**
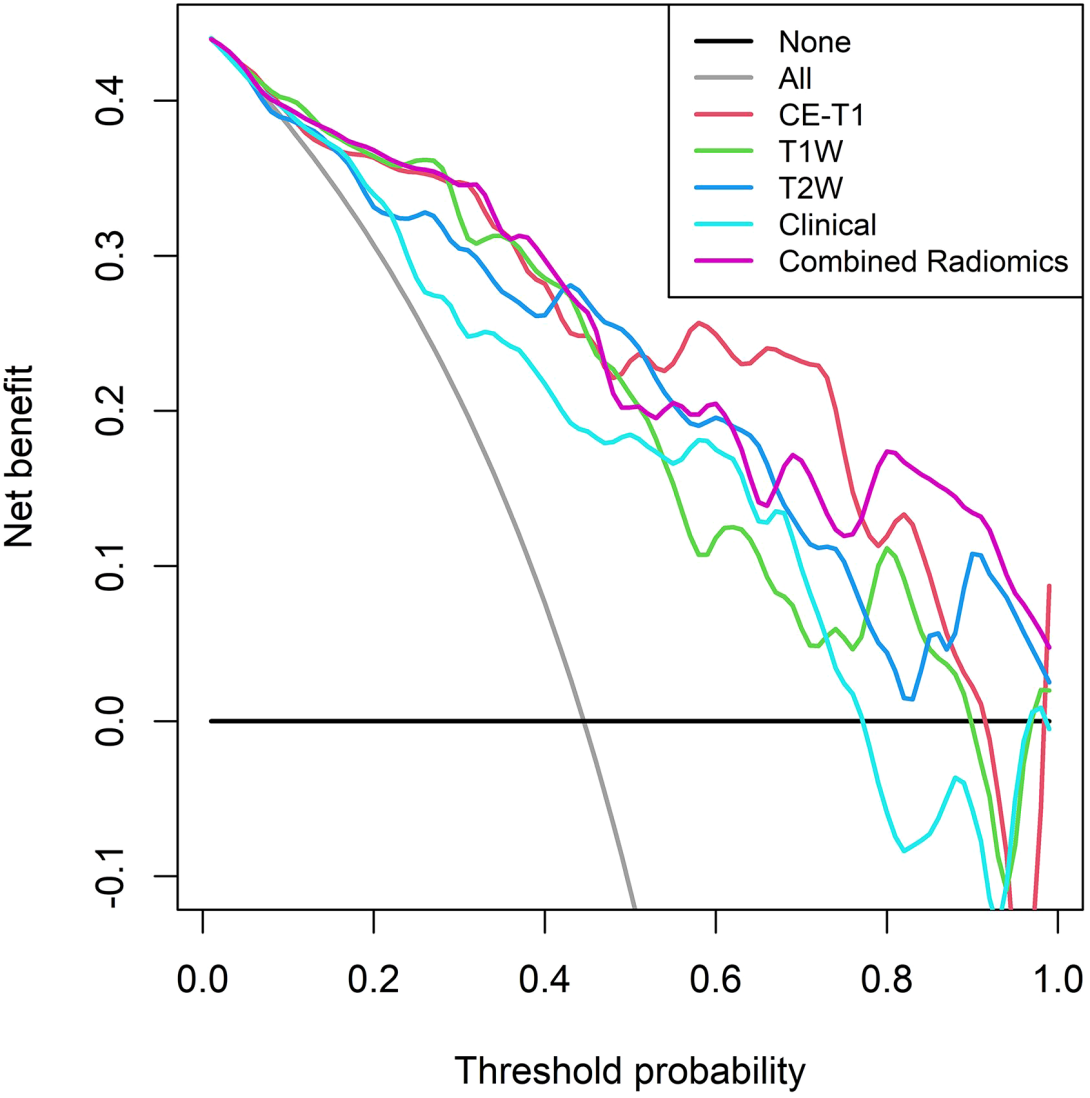
DCA of the training set shows that when the threshold probability is greater than 0.786, the net benefit of using the combined radiomics model to predict the invasion of PitNETs is higher.

The calibration curve shows that the prediction efficiency of the model is in good agreement with clinical observations (**Figure 6**). Additionally, DCA showed that when the threshold probability was greater than 0.786, the net benefit of using the combined radiomics model to predict the invasiveness of PitNETs was significantly higher than that of the other radiomics models (**Figure 7**).

## Discussion

In the present study, a predictive mpMRI-based radiomics signature model was established to predict the invasiveness of PitNETs and provide a preoperative individualized patient evaluation. The results of the present study showed that the predictive efficiency of the combined radiomics signature model was better than that of the clinical and single radiomics signature models, and could accurately and efficiently predict the invasiveness of PitNETs.

Previously, the evaluation of MRI-based Knosp grading played an important role in the analysis of the invasion of PitNETs into the CS, which is an important reference for the formulation of clinical treatment plans. Preoperative prediction of PitNETs invasiveness can assist in creating an individualized clinical treatment with precision medicinal treatment. Niu et al. (6) studied the preoperative prediction of the invasiveness of Knosp II and III PitNETs into the CS, based on CE-T1 and T2WI images. The AUCs of the nomogram established by combining radiomics features and clinical risk factors in the training and testing sets was 0.899 and 0.871, respectively, indicating a high prediction efficiency. In the invasive prediction model based on LR created in the present study, the AUCs of the combined radiomics signature model in the training and testing sets were 0.885 and 0.864, respectively. This model also had a high prediction efficiency. In contrast to previous studies, the present study divided the PitNETs into an invasive and a non-invasive group, which expanded the scope of tumor research and could be applied to all tumor patients with different Knosp grades. According to relevant literature (7), 25% of Knosp grade I PitNETs will extend into the parasellar region, while approximately 1.5% of these tumors are observed to have significant invasiveness into CS during surgery. Therefore, the present study included a wider range of subjects, and the results were more reliable and robust. Additionally, Liu et al. (23) conducted a texture analysis of preoperative DCE-MRIs to evaluate the vascular heterogeneity and invasiveness of PitNETs. The results of their analysis showed that the total model had the highest prediction efficiency (AUC = 0.957) and could effectively and accurately predict PitNETs invasion. It is worth noting that their study included fewer patients (n = 50), no validation set for the testing model, and the prediction model had the risk of overfitting. In the present study, the inclusion of data diversification prevented overfitting of the model, and the prediction results were, therefore, more stable and reliable.

Clinical demographic information statistics showed that there was a statistically significant difference in the maximum tumor diameter between the invasive and non-invasive groups (*P* < 0.01), indicating that tumor diameter was an important shape feature for distinguishing PitNETs invasiveness. Shape features are an important quantitative feature, as they can describe the shape and geometric characteristics of the ROI, such as volume, maximum diameter along different orthogonal directions, maximum surface area, tumor density, and sphericity (30). Additionally, some studies have not only outlined the tumor itself but also the surrounding peritumoral region. The research found that obtaining information about the peritumoral tissue can more accurately determine the tumor’s invasiveness to the surrounding tissues. This indicates that the radiomics information of the peritumoral region is important equally (31). In the present study, after feature screening, it was determined that the shape features showed a higher discrimination ability. The larger the tumor diameter and the more irregular its shape, the greater the possibility of invasion, which is consistent with the conclusion reached Liu et al. (23). In addition to the tumor shape, texture analysis, an image post-processing technique that uses representation algorithms to analyze the distribution and arrangement of all pixels in medical images and convert them into quantitative features (23, 24, 32–36), plays an important role in evaluating tumor heterogeneity. In the present study, a large number of texture features were extracted to establish the prediction models. It is worth noting that previous studies have confirmed that the classification performance of prediction models trained with multi-center data from different institutions is greatly reduced compared with the use of data from the same institution (37, 38). Therefore, multi-center research to improve model stability is very important and has clinical significance. Compared with previous single-center data sources and single-sequence extraction features used to establish prediction models (39, 40), the present study collected multi-center and diversified data, and through multi-dimensional feature extraction and mpMRI analysis, more quantitative tumor information can be obtained. The established combined radiomics signature model has higher prediction efficiency, better robustness, and versatility than previous models, and can be used in clinical multi-center applications to provide an objective and credible basis for treatment planning.

Like most studies, our study has several limitations: first, the study was a retrospective case collection, and there may be case selection bias, which requires prospective case inclusion in future studies; second, the number of cases in this study was relatively small, and rich clinical data could improve the performance of the prediction model; third, this study is a single-center study. Including more diverse dataset from multiple institutions could enhance the model’s generalizability, and we will further investigate this in the future; finally, manual segmentation of the tumor ROI is a time- and energy-consuming task, for which recent studies (25) have achieved significant success in automatically segmenting the background of the sellar region using deep-learning algorithms, an important direction for the future development of radiomics.

In conclusion, the mpMRI-based radiomics model is feasible for predicting the invasiveness of PitNETs before surgery, which can provide a basis for the clinical formulation of surgical plans and individualized treatment plans, improve the quality of life of patients after surgery, and mitigate postoperative tumor progression or recurrence, to a certain extent.

## Data Availability

The original contributions presented in the study are included in the article/supplementary material. Further inquiries can be directed to the corresponding author/s.
